# Alfalfa (*Medicago sativa*) Sprouts Respiratory Responses to Cadmium Stress Using IR LPAS

**DOI:** 10.3390/molecules27061891

**Published:** 2022-03-15

**Authors:** Cristina Popa, Mioara Petrus, Ana Maria Bratu

**Affiliations:** Laser Department, National Institute for Laser, Plasma and Radiation Physics, 409 Atomistilor St., P.O. Box MG-36, 077125 Magurele, Romania; mioara.petrus@inflpr.ro (M.P.); ana.magureanu@inflpr.ro (A.M.B.)

**Keywords:** alfalfa sprouts, ethylene plant hormone, ammonia, IR LPAS

## Abstract

Cadmium (Cd) is not considered a fundamental element for plants inducing general growth disturbances and inhibition in many species of plants. The purpose of our research was to examine the ethylene (C_2_H_4_) and ammonia (NH_3_), emissions in alfalfa sprouts with or without Cd, using infrared laser photoacoustic spectroscopy (IR LPAS), in order to suggest new markers that may add a better knowledge of Cd effect. The responses of alfalfa sprouts to C_2_H_4_ and NH_3_ may fluctuate, depending on tissue sensitivity and the phase of plant development. From the determinations of this study, the C_2_H_4_ was established to be inhibited, while NH_3_ was established to be in a higher concentration with the amount of Cd added to the alfalfa seeds for growth when the values were correlated to the control and BIOalfalfa sprouts (Sonnentor).

## 1. Introduction

Currently, plants are able to present a large number of metabolites either involved in the metabolic pathways or molecules characteristic for each plant which are a response to specific stimuli. Volatile organic compounds (VOCs) are secondary metabolites which are compounds generated and emitted by the plants under stress, such as Cd intake [1,2,3]. Through the years, different techniques have beenemployed to detect VOCs generated by living organisms under different stressors.

Gill et al. [3] discussed the regulatory mechanism of S uptake and assimilation, glutathione, and phytochelatins synthesis for Cd stress tolerance in crop plants using a variety of methods used to prevent heavy metals from affecting their growth.Ethylene is a regulatory substance produced and transported into the plant tissue taking part as a regulatory molecule in plant reactions and controlling plant responses under optimal and stressful circumstances [4,5]. The action of ethylene is notconducted only by internal ethylene produced in tissues, but also by the tissue sensitivity, and in general, is accepted that molecules involved in ethylene transduction it may control how much ethylene is required to excite a physiological response in plant [5,6,7].

In other research, Gill et al., 2011 [7] highlighted the alterations in morphological, physiological, and biochemical characteristics in crop plants under Cd stress together with the importance of plant mineral nutrients in the amelioration of Cd stress. Gill et al. [7] present a variety of methods used to prevent heavy metals from affecting their growth starting with the isolation of several miRNAs from rice, chelation, and sequestration of excess metal ions by binding with metal-chelating molecules such as metallothioneins, ferritin, or phytochelatins, or modulation in the ion transporters for influx or efflux of heavy-metal ions. These methods are only a few of the possible strategies for counteracting heavy metal stress.

In plant tissues, ammonium is produced in a lot of internal chemical activity, different researchers discovering that ammonium triggers more than one physiological and morphological reaction [1,8,9]. As many of these reactions are free of ammonium assimilation, ammonium itself reacts as a unit of a chemical compound that can take part in a chemical reaction IR LPAS-based trace gas detection demonstrated to be a promising technique that has been extensively useful to the study of living organisms, particularly due to its high sensitivity and selectivity, but also easily reproducible protocols [8,9,10,11,12,13,14,15].

This investigation debates the study of gases signal for ethylene and ammonia released by alfalfa sprouts to Cd effect in the presence of synthetic air and using IR LPAS method.

## 2. Results

This analysis examines the ethylene and ammonia responses in alfalfa a germinated with distilled water and used as the control and BIO alfalfa sprouts compared with alfalfa germinated with 3 mL of Cd and alfalfa germinated with 6 mL of Cd. BIO samples are alfalfa sprouts purchased from retail outlets with organic seeds sprouted in varying proportions while control samples are given by alfalfa seeds from certified organic farming germinated with distilled water in the laboratory at room temperature. All the biological samples were analyzed in a synthetic air environment at room temperature conditions using IR LPAS.

**Figure 1:**
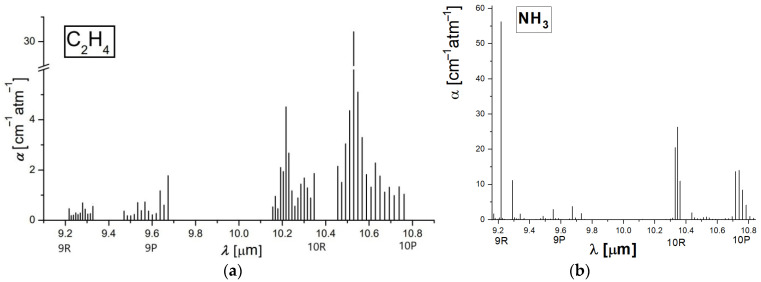
The absorption coefficients at laser lines of (**a**) ethylene (**b**) ammonia [10,11,14].

Taking into account that the number of the absorbing molecules is proportional to the amplitude of the signal, ethylene and ammonia emission was established for 6 g of alfalfa sprouts into the biological sample for approximately 3600 s. A chemical compound that absorbs laser radiation is stimulated to a higher quantum condition causing a reduction in laser light intensity, which can be directly quantified via absorption spectroscopy [11,15]. The absorption characteristics distinctive to each chemical compound make it feasible to detect trace gases and establish their accumulations. Absorption factors (see Figure 1) are in most cases in the order of 1 cm^−1^. To improve the quantification of ethylene and ammonia absorption coefficients, an adjusted process was adopted.

To determine gas absorptions, it is imperative to calibrate the resonant cylindrical cell with a well-known gas compound and to establish the linearity of the detector with the concentration of the known gas over orders of magnitude. The linear responses of the cavity for low detection limit of ethylene and ammonia in literature is reported [10,11,12,13,14,15].

For the ethylene absorption coefficients determination (see Figure 1a) at laser lines, we used a commercially prepared, certified mixture containing 0.96 ppm ethylene in pure nitrogen. We examined this mixture at a total pressure of approximately 1030 mbar and a temperature T ≅ 23 °C using the accepted value [10,11,12,13,14,15] of the absorption coefficient of 30.4 cm^−1^ atm^−1^ at the 10P(14) line of the laser (10.53 μm).

In the case of ammonia absorption coefficients determination (see Figure 1b), we used a certified mixture containing 10 ppm of ammonia in pure nitrogen. We examined this mixture at a total pressure of approximately 1030 mbar and a temperature T ≅ 23 °C, using the value of the absorption coefficient of 57.12 cm^−1^ atm^−1^ at 9R(30) laser transition (9.22 μm) [10,11,14].

So long as the absorption factors of ethylene and ammonia at separate laser wavelengths were specifically estimated [10,11,12,13,14,15], the CO_2_ laser was set at specific lines; first on the 10P (14) line and then on the 9R(30) line.

**Figure 2:**
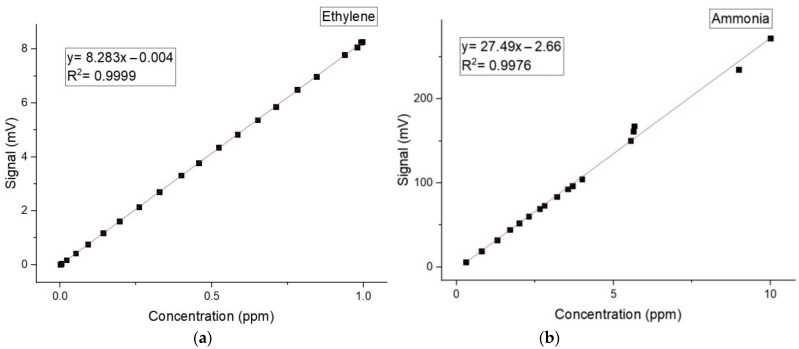
The concentration-dependent response for (**a**) ethylene and (**b**) ammonia.

The calibration measurements (concentration-dependent response) for both ammonia and ethylene (see Figure 2a,b) were experimentally determined using the accepted values for ethylene and ammonia [10,11,12,13,14,15].

Table 1 shows the measurable factors that were available by this application for the determination of the gas molecules in alfalfa sprouts.

For the ethylene and ammonia determination, we use software that records on different panels both the laser power, photoacoustic signal, and trace gases concentration.

**Figure 3:**
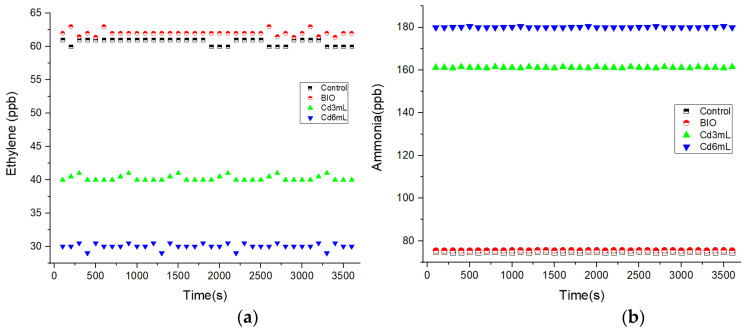
The alfalfa sprouts respiratory responses to Cd stress using IR LPAS spectroscopy for alfalfa germinated with distilled water (control), correlated with BIO alfalfa sprouts and with alfalfa germinated with 3 and 6 mL of Cd; (**a**) ethylene, (**b**) ammonia.

Figure 3 presents the alfalfa sprouts respiratory responses to Cd stress using IR LPAS. Although the fluctuation of laser power is insignificant, we want to add that the fluctuations on the measurements presented in Figure 3a are explained by the sensitivity which is limited by laser power fluctuations.

By increasing the amount of Cd (from 3 to 6 mL), for germinated alfalfa seeds (see Figure 2a), the concentration of ethylene was inhibited from 40 to 30 ppb compared with the control (60.7 and 62 ppb).

In contrast to Figure 3a, when analyzing Figure 3b, the ammonia emissions increase with the increasing amount of Cd from 161 (for 3 mL of Cd) to 180 ppb (for 6 mL of Cd) with the correlation of the results with the control (75 and 75.5 ppb).

From the obtained results, the rate of the trace gas ethylene was inhibited along with the increase in the amount of Cd, whereas the rates for the trace gas ammonia become greater along with the increase in the amount of Cd.

## 3. Discussion

A number of studies [16,17,18,19] have shown that Cd significantly affects the plant growth compared with other several studies [20] showing that the plant is tolerant to the stress produced by Cd.

Sharmila et al. [18] elucidated the key factor behind Cd^2+^-toxicity-induced proline accumulation in Indian mustard by raising seedlings, independently in distilled water and mineral growth medium in the presence of 0–500 μM CdCl_2_. The results convincingly demonstrated that Cd^2+^-induced iron deficiency promotes proline accumulation.

Yoshihara et al. [19] present the responses induced by Cd exposure in plants using macro and molecular indices. The results demonstrated the responses of Cd-inducible by Fe-deficiency at a molecular level.

Compared with [18,19] studies, Honghua et al. [20] present a pot experiment to investigate the individual and combined effects of Cd and Pb level in a calcareous soil on the status of mineral nutrients, including K, P, Ca, Mg, S, Fe, Mn, Cu, and Zn, in alfalfa (*Medicago sativa* L.) plants. The results indicate that alfalfa is tolerant to Cd and Pb stress, and it is promising to grow alfalfa for phytostabilization of Cd and Pb on calcareous soils contaminated with Cd and Pb.

As with Kabir et al. [21], the results of the present study showed that Cd stressed the alfalfa plants, as it is associated with the rise of the free radicals and reactive oxygen species generation [22].

While all the studies presented above [16,17,18,19,20,21,22] analyze the internal of plants, the study from this manuscript presents novelty by analyzing the alfalfa respiration using IR LPAS technique in the assessment of the trace gases.

The running analysis depicts an experimental determination of ethylene and ammonia liberated by alfalfa plants using seeds germinated with or without Cd, with help of IR LPAS. These evaluations are achieved in various environmental conditions in a direct knowing of the change, activity, or progress of plants respiration. Although Cd is a toxic trace element which is not essential for plants, this can be easily taken up by roots and accumulated in various organs, and cause irreversible damages to plants, having a stable impact on ethylene and ammonia signaling. It is admissible to roughly calculate that ethylene and ammonia has a definitive contribution, since it is known that ethylene is a senescence hormone and ammonia is a signaling molecule.

From the present data regarding the effect of Cd on alfalfa sprouts, seems that the alfalfa sprouts were affected by the amount of heavy metal added to the germination. The ethylene is repressed while the ammonia is increased related to the gases from the alfalfa sprouts signal.

The present results make sure that other investigations [16,17,18,19,20,21,22] are accurate indicating that the Cd at plants makes a difference in the respiration process for ethylene and ammonia with negative effects on the alfalfa sprouts.Cd can restrain the alfalfa sprouts’ progress, which is correlated with the physiological and biochemical state of the plant. The analysis is determined by the ethylene and ammonia emission from the respiration of plants in stress conditions using IR LPAS and is in proper accord with those presented in the scientific literature [16,17,18,19,20,21,22].

To measure the ethylene and ammonia from the biological samples, the software user interface was modified to allow the laser power, photoacoustic signal, and calculated gases concentration function on time to be recorded on different panels.

## 4. Materials and Methods

**Figure 4:**
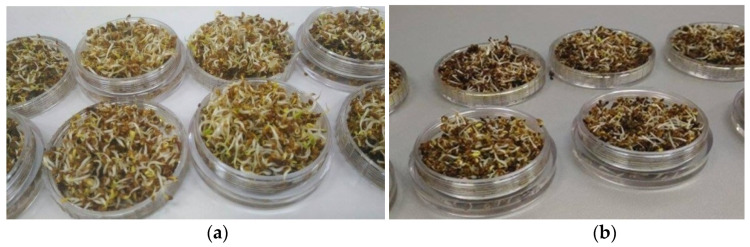
Capsules used for alfalfa sprouts: (**a**) Alfalfa seeds germinated with distilled water (used as control); (**b**) alfalfa seeds germinated with Cd.

The research was devoted to evaluating the Cd tolerance in the detection of ethylene and ammonia in alfalfa sprouts testing CO_2_ laser photoacoustic spectroscopy method with respect to the CO_2_ laser frequencies [9,10,11,14,23,24]. For the experimental analysis, we used ~6 g of alfalfa seeds for every sample and the germination was carried out at room temperature. The seeds were grown in colorless polycarbonate containers (see Figure 4) with a specific volume of 0.83 cm³/g volume [25,26]. In each small container were added 6 g of alfalfa seeds over which we added 10 mL of distilled water for control samples or Cd for treated samples. The solutions were replenished every day. After the germination, the containers were inserted into a glass cuvette that was inserted in the experimental set-up for the identification of ethylene and ammonia presence [1,24]. All data are expressed as a mean from at least 3 independent experiments (3 experiments × 3 replicates).

Cd was procured from the Research and Development National Institute for Metals and Radioactive Resources, Bucharest, Romania, and had a concentration of Cd 2 µM, 0.2248 mg/L (initial standard Merck METAL 1 g/L: CdCl_2_, 99.99% trace metals basis dissolved in pure water) [9]. Because Cd is a heavy metal that is taken up and stored faster than is metabolized or excreted, the experimental determinations of plantlets for alfalfa were chosen to be done about 3–4 days, after the germination of the seeds for a period of about 3600 s.

**Figure 5:**
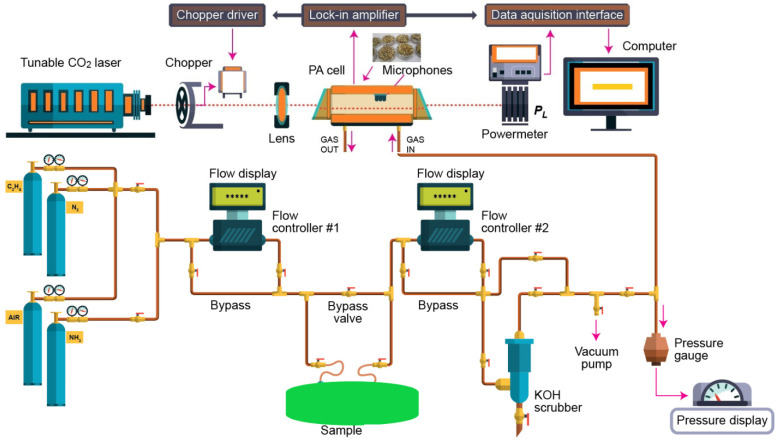
IR LPAS set-up used for the evaluation of ethylene and ammonia.

The CO_2_ laser photoacoustic spectroscopy set-up used for the evaluation of the ethylene and ammonia is presented in Figure 5 and more in detail described in [1,9,10,11,14,23,24,25].

This technique operates on the principle that the amount of light absorbed by a sample is related to the concentration of the target species in the sample. Light of known intensity is directed through a gas sample cell and the amount of light absorbed by the sample is measured as a sound intensity by a detector, usually a sensitive microphone.

V = *α*CS*_M_*P*_L_c*(1), (1)

where V (V) is the photoacoustic signal; *α* (cm^−1^ atm^−1^), the gas absorption coefficient at a given wavelength; C (Pa cm W^−1^), the cell constant; S*_M_* (V Pa^−1^), the microphone responsivity; P*_L_* (W), the cw laser power; and *c* (atm), the trace gas concentration (usually given in units of per cent, ppmV, ppbV, or pptV).

According to this equation, the photoacoustic signal is linearly dependent on the absorption coefficient, cell constant, microphone responsivity, incident laser power, and absorbent trace gas concentration, which means that this technique is a “zero-baseline” approach since no signal will be generated if the target molecules are not present.The photoacoustic arrangement consists of a homemade CO_2_ laser, a lens, a chopper (model DigiRad C-980, Terahertz Technologies Inc., Oriskany, New York, NY, USA), a photoacoustic cavity, a powermeter (model Rk-5720, Laser Probe Inc., Utica, NY, USA), a lock-in amplifier (model SR830, Stanford Research Systems, Sunnyvale, CA, USA), an acquisition panel (TestPoint software, Keithley, Cleveland, OH, USA), and a data-processing computer. The experimental detection system also includes a gas handling system with an essential role in the control of the studied gases, but it can also perform other actions described in detail in other articles [23,24,25].

Basically, an IR LPAS system uses an adjustable laser and a H-type resonant cylindrical cell where the gas is detected. The laser beam entering the cylindrical cell after modulating and focusing.

The sample was enclosed in a cylindrical cell, where the acoustic waves are detected by four miniature microphones (Knowles Acoustics, model EK 3033, Itasca, IL, USA) connected in series. The microphones turned the acoustic signal into an electrical signal that is detected by a lock-in amplifier with a role in setting the chopper frequency and providing the photoacoustic signal. After a passage through the stainless steel and Teflon cylindrical cell, the power of the laser beam was measured by a laser radiometer. Its digital output was introduced in the data acquisition interface module together with the output from the lock-in amplifier. The acquisition and processing of the recorded data were done with Keithley TestPoint software. TestPoint data acquisition software provided a development environment in which data acquisition applications can be generated. The light beam was modulated with a high quality, low vibration noise, and variable speed between 4–4000 Hz, mechanical chopper with 30-slot aperture, operated at the appropriate resonant frequency of the H-type resonant cylindrical cell (564 Hz). The diverging infrared laser beam was converged by a ZnSe focusing lens (*f* = 400 mm). In this way, a slightly focused laser beam was passed through the resonant cylindrical cell without wall interactions. All experimental data were processed and stored by a computer. More details about the experimental system can be found in the articles already published [1,9,10,11,14,23,24,25,26].

It has to be underlined that the measured photoacoustic signal is due mainly to the absorption of gases, but some traces of CO_2_, H_2_O, ethanol, etc., influenced the measurements. The overall contribution was <10%. To examine the alfalfa sprouts’ signal response from the samples, we inserted a KOH trap (with a volume >100 cm^3^) in order to eliminate the interference of other gases (such as CO_2_ and H_2_O).The response to all absorbing species at a given laser wavelength decreased considerably when we inserted a KOH trap, proving that amounts of CO_2_ and H_2_O vapors in the samples can significantly alter the results, thus making their removal compulsory [27].

To examine the alfalfa sprouts’ signal response for 3 and 6 mL of Cd from the samples, we removed the extra gas, and then cleaned the circuit with pure nitrogen at atmospheric pressure for a few minutes. To guarantee accurate determinations, a vigorous cleaning was accomplished between every biological test.

After the system was flushed, we transferred the gas from the sample by using a synthetic air flow near atmospheric pressure (1030 mb).

## 5. Conclusions

Careful investigation of the ethylene and ammonia signals at alfalfa seeds germinated with or without Cd was done in the presence of the synthetic air flow at atmospheric pressure using IR LPAS.

From the investigations, the ethylene at alfalfa sprouts was established in lower concentrations whereas ammonia molecules were established in higher concentrations.

As a result, this study promotes the findings that ethylene and ammonia in alfalfa respiration may present as essential in plant progress and development.

With a fully non-invasive procedure, the IR LPAS technique has the potential to separate alfalfa sprouts with or without Cd.

IR LPAS technique plays an important role in assessing the concentration of ethylene and ammonia released by the alfalfa sprouts subject to the Cd effect. The attributes associated with the IR LPAS technique are sensitivity, selectivity, versatility, reliability, robustness, and ease of use. With improved sensitivity and specificity, IR LPAS technique analyses of biological samples might offer a new approach to the detection of gases and a better understanding of the metabolic basis of alfalfa sprouts.

## Figures and Tables

**Table 1:** Selecting measurable factors for gases determination in alfalfa sprouts.

Measurable Factors	Values
Ethylene certified mixture for measurement of ethylene absorption coefficients	0.96 ppm ethylene in pure nitrogen
Ammonia certified mixture for measurement of ammonia absorption coefficients	10 ppm ammonia in pure nitrogen
CO_2_ laser wavelengths	9–11 µm
*α* (cm^−1^ atm^−1^): the gas absorption coefficient at a given wavelength;	V = *α*CS*_M_*P_L_*c*
Cylindrical chamber cell pressure	≈1030 mb
The total quantity of alfalfa sprouts	≈6 g
Cd concentration	2 μM–0.2248 mg/L
Gas absorption coefficient	ethylene-10P(14); λ = 10.53 μm; *α* = 30.4 cm^−1^ atm^−1^ ammonia-9R(30); λ = 9.22 μm; *α* = 57 cm^−1^ atm^−1^
Synthetic air outflow composition	Linde gas: 20% oxygen, 80% nitrogen (impurities: hydrocarbons max. 0.1 ppmV, nitrogen oxides max. 0.1 ppmV)
Temperature	≈23–25 °C
Glass sample volume	150 cm^3^
Cylindrical chamber cell volume	1000 cm^3^
Responsivity	375 cmV/W
Alfalfa time analysis	≈3600 s

## Data Availability

Not applicable.

## Sample Availability

Samples of the compounds are available from the authors.
